# Minding the treatment gap: results of the Singapore Mental Health Study

**DOI:** 10.1007/s00127-019-01748-0

**Published:** 2019-07-17

**Authors:** Mythily Subramaniam, Edimansyah Abdin, Janhavi Ajit Vaingankar, Saleha Shafie, Hong Choon Chua, Weng Mooi Tan, Kelvin Bryan Tan, Swapna Verma, Derrick Heng, Siow Ann Chong

**Affiliations:** 1 Research Division, Institute of Mental Health, Buangkok Green Medical Park, 10 Buangkok View, Singapore, 539747 Singapore; 2 Lee Kong Chian School of Medicine, Singapore, Singapore; 3 CEO Office, Institute of Mental Health, Singapore, Singapore; 4 Community Mental Health Division, Agency for Integrated Care (AIC), Singapore, Singapore; 5 Policy, Research and Evaluation Division, Ministry of Health, Singapore, Singapore; 6 Department of Psychosis and East Region, Institute of Mental Health, Singapore, Singapore; 7 Epidemiology and Disease Control Division, Ministry of Health, Singapore, Singapore

**Keywords:** Major depressive disorder, Generalized anxiety disorder, Obsessive compulsive disorder, Alcohol use disorder, Prevalence

## Abstract

**Purpose:**

To establish the 12-month treatment gap and its associated factors among adults with mental disorders in the Singapore resident population using data from the second Singapore Mental Health Study and to examine the changes since the last mental health survey conducted in 2010.

**Methods:**

6126 respondents were administered selected modules of the Composite International Diagnostic Interview, to assess major depressive disorder (MDD), dysthymia, bipolar disorder, generalized anxiety disorder (GAD), obsessive compulsive disorder (OCD) and alcohol use disorder (AUD) (which included alcohol abuse and dependence). Past year treatment gap was defined as the absolute difference between the prevalence of a particular mental disorder in the past 12 months preceding the interview and those who had received treatment for that disorder.

**Results:**

The prevalence of overall 12-month treatment gap in this population was high (78.6%). A multiple logistic regression analysis revealed significantly higher odds of treatment gap among those diagnosed with OCD (compared to those with MDD) and in those with a comorbid chronic physical disorder; while those who had primary education and below and those who were unemployed were less likely to have a treatment gap as compared to those with post-secondary education and those employed, respectively.

**Conclusions:**

The high treatment gap in the population is concerning and highlights the need to promote help-seeking and uptake of treatment. Given the unique demographic characteristics, i.e., those with higher education and employed were more likely not to seek treatment, targeted interventions in the educational and workplace settings should be implemented.

**Electronic supplementary material:**

The online version of this article (10.1007/s00127-019-01748-0) contains supplementary material, which is available to authorized users.

## Introduction

The World Health Organization’s global health report has estimated that 4.4% of the world’s population suffers from depression and 3.6% from anxiety disorders which means that globally 300 million suffer from depression and 264 million from anxiety disorders [1]. Given the high prevalence, chronic nature and comorbidity with both physical and mental disorders, mental disorders are associated with significant disability. The Global Burden of Diseases Study reported that mental and behavioral disorders accounted for 22.7% of all years lived with disability (YLDs) globally and that neuropsychiatric disorders were the leading cause of global YLDs [2]. However, there is a significant ‘treatment gap’, i.e., the difference between the number of individuals in need of mental health care and those who actually receive treatment [3]. The World Mental Health Surveys (WMHS) found that few people received treatment for their emotional or substance use problems in the 12-month period prior to the survey. It ranged from a low of 0.8% in Nigeria to a high of 15.3% in the United States [4].

Evidence increasingly shows that common mental disorders like depression and anxiety can be diagnosed and treated using medications and psychological therapies even in primary care settings [5, 6]. Yet, a number of factors adversely affect help-seeking; these include: people wanting to deal with the problem on their own [7], lack of knowledge of the condition [8], stigma [9, 10], lack of accessibility which includes cost, lack of transportation and scheduling [11, 12], and availability barriers including long wait time, lack of services and staff [13]. This treatment gap affects not only the persons with mental disorders but also their families, employers and governments. Consequences for the individual with untreated mental disorder include increased contact with the criminal justice system, reduced levels of employment and often lower salaries when employed, personal relationship difficulties, lack of attention to physical health problems and premature death [14–16]. At the societal level, untreated mental disorders result in reduced productivity, increased healthcare and other welfare expenditures [17, 18].

In a recent study by Font et al. [19], lifetime treatment gap was found to be positively associated with being male and practicing religion, and negatively associated with aging, being single, being a French native (versus other nationalities) and experiencing a mood, anxiety, alcohol or substance use and psychotic disorder (versus those not having any disorder). In contrast, a study by Michel et al. [20] that examined current help-seeking found that functional impairment and presence of an affective disorder were associated with the highest odds of help-seeking. Other sociodemographic variables, such as age and gender, were unrelated to current help-seeking. Evans-Lacko et al. [21] analyzed data from the WHO world mental health surveys and found no significant association of income with overall treatment in the total sample, while education was most consistently associated with specialty mental health treatment, but the associations with treatment in other service sectors were relatively weak. Data from these studies, thus, suggest that factors associated with help-seeking, and therefore treatment gap differs across populations and settings.

A population-based epidemiological study done in Singapore in 2010 established that significant treatment gaps existed across mood, anxiety and alcohol use disorder [22]. In the past 10 years, the Singapore government has implemented a number of mental health policies and programs as part of its continuing efforts to reduce the treatment gap and disease burden. The community mental health master plan was launched in 2012, and underwent an expansion phase from 2015. The main focus of this master plan was to strengthen primary care to improve access to mental health services and to integrate and expand pre- and post-treatment support in the community to ensure integrated delivery of care. This multi-faceted plan adopted a population health approach that included creating awareness and empowering the community via building local networks, providing early assessments and interventions in the community, referral to specialized services as and when needed, and step-down care and rehabilitation in the community. The goals of this plan were to create an inclusive community and provide person-centric and integrated support across healthcare and social sectors. We were, therefore, particularly interested in examining the early effects of this master plan and if there were any changes in the treatment gap since its implementation.

Thus, the aims of the current study were to establish the 12-month treatment gap and its associated factors among adults with mental disorders in the Singapore resident population using data from the second Singapore Mental Health Study and to examine the changes since the last mental health survey which was conducted in 2010 (henceforth referred to as SMHS 2010).

## Methods

### Sample

The second SMHS conducted in 2016, (henceforth referred to as SMHS 2016) was a cross-sectional, population-based, epidemiological study [23]. Face-to-face interviews were conducted with eligible respondents over a 1-year period. In all, 6126 Singapore citizens and permanent residents participated in the study and represented the resident population aged 18 years and older.

The study was approved by the National Healthcare Group’s Domain Specific Review Board. Written informed consent was obtained from all participants and parents or legally acceptable representatives of those aged below 21 years.

### Assessments

#### World Mental Health Composite International Diagnostic Interview (WMH-CIDI)

All the respondents were administered the WMH-CIDI [24], a fully structured diagnostic interview to assess the disorders and their treatment. Diagnostic modules for lifetime and 12-month prevalence of mood disorders, including major depressive disorder (MDD), dysthymia and bipolar disorder; anxiety disorders, including generalized anxiety disorder (GAD) and obsessive–compulsive disorder (OCD); alcohol use disorders (AUD), i.e., alcohol abuse and dependence were included. Disorders were assessed using the definitions and criteria of the Diagnostic and Statistical Manual of Mental Disorders, Fourth Edition (DSM-IV) [25]. CIDI organic exclusion rules were applied to all diagnoses. Age of onset of each disorder was collected and classified as adolescent (11–17 years), young (18–35), and older (36–68) onset.

### Treatment Gap

The outcome variable was past-year treatment gap which was defined as the absolute difference between the prevalence of a particular mental disorder in the past 12 months preceding the interview and those who had received treatment for that disorder, i.e., the percentage of those who needed but were not receiving treatment. It was ascertained in two ways: (i) in each WMH-CIDI diagnostic module, respondents were asked a series of questions to establish if they had ever sought treatment. They were first asked whether they had ever in their life ‘talked to a medical doctor or other professional’ about the disorder. The term ‘other professional’ is defined by WMH-CIDI as a wide range of professionals, including psychologists, counselors, spiritual advisors, herbalists, acupuncturists, and any other healing professionals. Those who stated that they had received treatment were further probed if they had received professional treatment at any time in the past 12 months, (ii) 12-month treatment was also assessed through the ‘services’ module by asking respondents if they ever saw any of the professionals from a predetermined list for problems with emotions, nerves, mental health, or use of alcohol or drugs. Included were mental health professionals (e.g., psychiatrist, psychologist), general medical professionals (e.g., general practitioner, family physician), other specialist medical doctors (e.g., cardiologist, gynecologist), other mental health professionals (e.g., nurse, occupational therapist), religious advisors (e.g., minister, priest), and traditional healers (e.g., herbalist, chiropractor).

Barriers to seeking care were elicited from the ‘services’ module of the WMH-CIDI. Those respondents who had not sought help despite feeling that they needed help for at least a period of 4 weeks were asked the following question—‘Here are some reasons people have for not seeking help even when they think they might need it. Just tell me “yes” or “no” whether each statement applies to why you did not see a professional in the past 12-months’.

### Sheehan Disability Scale (SDS)

The SDS [26] was used to assess the level of interference in four life domains (home management; ability to work; ability to form and maintain close relationships with other people; and social life) during the worst period of symptoms in the 12 months prior to interview. An overall level of impairment was determined for each disorder. A disorder was classified as serious if the respondent reported at least three areas of role functioning with severe role impairment due to the mental disorder. A disorder was classified as mild if the interference was rated as at least two areas of role functioning with mild in any SDS domain. All other disorders were classified as moderate [27].

### Socio-demographic data

Data on gender, age groups (18–34 years, 35–49 years, 50–64 years, and 65 years old and above), ethnicity (Chinese, Malay, Indian, and Others), marital status (single, married, divorced/separated or widowed), educational level (primary and below, secondary including vocational/ITE, and post-secondary including pre-university/junior college/diploma and university), employment status (employed, unemployed and economically inactive, i.e., students, homemakers and retirees) and household income (3999 Singapore Dollar (SGD) and below, SGD 4000–5999, SGD 6000 and above per month) were collected. Household income was calculated as the sum of all pre-tax income in the past 12 months, of all family members living in the same household and divided by 12 to derive the monthly income.

### Statistical analysis

All estimates were weighted to adjust for over sampling, non-response and post-stratified for age and ethnicity distributions between the survey sample and the Singapore resident population in 2014. Descriptive analyses were performed to establish the 12-month treatment gap of mental disorders as well as describe the socio-demographic profile of the study population. Socio-demographic correlates of treatment gap were examined using backward and forward stepwise logistic regression analyses. To compare the rates of treatment gap between the two surveys, data from SMHS-2016 were reanalyzed using similar sampling weight as the SMHS-2010 study. Significant differences in prevalence and severity were tested using Chi-square tests. Standard errors (SE) and significance tests were estimated using the Taylor series linearization method. All statistical analyses were performed using the Statistical Analysis Software (SAS) system version 9.3 (Cary, NC, USA).

## Results

A total of 6126 respondents were interviewed which yielded a response rate of 69.5%. Table 1 shows the demographic characteristics of the total sample. The weighted prevalence of any DSM-IV disorder in the past 12 months was 6.5%. More details of the individual disorders and their correlates have been reported in an earlier article [23]. Table 2 shows the proportion of those who met the criteria for 12-month mental disorder, but had not sought any treatment at the point of the survey (treatment gap), as well as the breakdown by severity. The overall treatment gap was high (78.6%), with alcohol dependence having the highest treatment gap (97%) followed by OCD (84%) and alcohol abuse (80.6%). The treatment gap for other disorders was—77.6% for bipolar disorder, 73% for MDD, 69.5% for dysthymia, with the lowest treatment gap observed among those with GAD (62.1%). Majority of the respondents who had not sought treatment met criteria for a mild disorder (68.2%) using the SDS criteria. Univariate analyses showed that the prevalence of treatment gap was significantly higher among those employed as compared to those who were unemployed (81.9% vs. 56.9%, *p* = 0.036), those with mild symptoms as compared to those with moderate/severe symptoms (83.5% vs. 67.1%, *p* = 0.0155) and those diagnosed with OCD as compared to those with MDD (88.3% vs. 73.4% *p* = 0.0076) (Supplementary Table 1). After controlling for all covariates in multiple logistic regression analysis, we observed significantly higher odds of treatment gap among those diagnosed with OCD (compared to those with MDD) and in those with a comorbid chronic physical disorder (compared to those without). Surprisingly, we found a progressive socio-economic status gradient, with the primary-educated and unemployed, having a lower treatment gap compared to the tertiary-educated and employed, respectively (Table 3).

**Table 1:** Socio-demographic distribution of the sample (N = 6126)

	*N*	Unweighted %	Weighted %
Age group (years) (Mean = 45.2)			
18–34	1707	27.9	30.4
35–49	1496	24.4	29.6
50–64	1626	26.5	26.9
65 +	1297	21.2	13.1
Gender			
Female	3058	49.9	50.4
Male	3068	50.1	49.6
Ethnicity			
Chinese	1782	29.1	75.7
Malay	1990	32.5	12.5
Indian	1844	30.1	8.7
Others	510	8.3	3.1
Marital status			
Never married	1544	25.2	31.0
Married	3843	62.7	59.8
Divorced/separated	343	5.6	5.2
Widowed	396	6.5	4.1
Education			
Primary and below	1187	19.4	16.3
Secondary	1648	26.9	23.0
Pre-U/Junior College	304	5.0	6.0
Vocational/ITE	508	8.3	6.3
Diploma	1024	16.7	19.0
University	1455	23.8	29.4
Employment			
Employed	4055	66.2	72.0
Economically inactive^a^	1716	28.0	22.7
Unemployed	354	5.8	5.3
Household income (SGD/month)			
Below 2000	1147	21.0	16.5
2000–3999	1331	24.4	20.0
4000–5999	1113	20.4	21.4
6000–9999	1003	18.4	21.8
10,000 and above	861	15.8	20.3
Severity^b^			
Mild	231	62.8	63.2
Moderate/severe	137	37.2	36.8
12-month CIDI^b^			
MDD	89	22.1	25.9
Dysthymia	5	1.2	1.4
Bipolar	38	9.4	6.2
GAD	29	7.2	7.5
OCD	124	30.8	30.6
AUD	36	9.0	8.4
Comorbidity (≥ 2 mental disorders)	82	20.4	20

*ITE* Institute of Technical Education, *SGD* Singapore Dollars

^a^Includes homemakers, students and retirees/pensioners

^b^The proportion was among those diagnosed with 12-month mental disorders only (N = 403)

**Table 2:** 12-month prevalence and severity in treatment gap in the SMHS-2016 survey

Disorder	Prevalence		Treatment gap		Treatment gap by severity of SDS			*p* value^#^
	*n*	%	*n*	%	Mild (%)	Moderate (%)	Severe (%)	
MDD	141	2.3	109	73.0	52.7	45.6	1.6	0.195
Dysthymia	21	0.2	14	69.5	50.0	50.0	–	0.276
Bipolar disorder	64	0.9	51	77.6	27.3	72.7		0.227
GAD	53	0.8	33	62.1	58.9	41.1	–	0.272
OCD	169	2.9	146	84.0	86.4	13.6	–	0.087
Alcohol abuse	33	0.6	28	80.6	98.5	1.5	–	0.0002
Alcohol dependence	18	0.2	17	97.0	89.1	1.1	–	–
Any mental disorder	403	6.5	328	78.6	68.2	32.3	5.5	0.0009

*Any mental disorder* has at least one of the mental illnesses assessed by the composite international diagnostic interview in the study, *GAD* Generalized anxiety disorder, *MDD* major depressive disorder, *OCD* obsessive compulsive disorder, *SDS* Sheehan Disability Scale

Statistical significance was evaluated at the *p* value < 0.05

^#^Chi-Square test; Chi-Square test was not estimated in alcohol dependence due to low numbers

**Table 3:** Sociodemographic correlates of treatment gap for 12-month mental disorders

	Multiple logistic regression analyses			
	OR	95% CI		*p* value
Age group				
18–34 (reference)				
35–49	0.3	0.1	1.3	0.107
50–64	1.2	0.2	6.0	0.810
65 +	0.5	0.03	8.1	0.608
Gender				
Female (reference)				
Male	1.7	0.5	5.1	0.380
Ethnicity				
Chinese (reference)				
Malay	1.5	0.5	4.7	0.518
Indian	0.8	0.3	2.3	0.654
Others	0.8	0.2	6.4	0.871
Marital				
Married (reference)				
Never married	3.0	0.7	13.5	0.157
Divorced/separated	1.0	0.2	4.7	0.975
Widowed	2.7	0.1	97.0	0.579
Education				
Post-secondary (reference)				
Primary and below	0.1	0.01	0.9	0.045
Secondary	1.0	0.3	3.1	0.997
Employment				
Employed (reference)				
Economically inactive	0.9	0.2	4.5	0.977
Unemployed	0.2	0.1	0.7	0.017
Income (SGD/month)				
Below 3999 (reference)				
4000–5999	0.9	0.2	4.0	0.855
6000 and above	0.4	0.1	1.3	0.126
Any chronic physical disorder				
No (reference)				
Yes	2.7	1.03	6.8	0.043
Severity				
Mild (reference)				
Moderate/severe	0.5	0.2	1.3	0.154
Age of onset				
Adolescence (reference)				
Young	0.8	0.2	2.6	0.676
Middle age and older	1.1	0.3	4.5	0.916
12-month CIDI				
MDD (reference)				
Dysthymia	0.9	0.1	8.5	0.961
Bipolar	1.9	0.3	13.2	0.537
GAD	0.5	0.1	3.1	0.434
OCD	6.3	1.4	28.9	0.017
AUD^a^	–	–	–	–
Comorbidity	1.1	0.3	4.7	0.906

Secondary education includes secondary and vocational education, post-secondary education includes pre-university or junior college, diploma, and university education; comorbidity refers to two or more mental disorders in the past 12 months

*Any mental disorder* has at least one of the mental illnesses assessed by the composite international diagnostic interviewin the study, *AUD* alcohol use disorder, *GAD* generalized anxiety disorder, *MDD* major depressive disorder, *OCD* obsessive compulsive disorder, *SGD* Singapore Dollar

^a^Not estimated due low number

Comparing the prevalence of treatment gap in SMHS 2016 to that observed in SMHS 2010 we found that the 12-month prevalence of treatment gap had decreased overall by about 4% from 82.1% to 78.4%. The treatment gap for alcohol abuse decreased significantly (80.5% from 96.9%, *p* = 0.007) and while there was a decrease in the 12-month treatment gap of MDD, bipolar disorder and GAD, it was not significant (*p* > 0.05) (Table 4).

**Table 4:** Comparison of 12-month prevalence of treatment gap in the SMHS-2010 and the SMHS-2016 surveys

Disorder	Treatment gap		Risk ratio	*p* value^#^
	2010 (%)	2016 (%)*		
MDD	75.3	72.8	0.97	0.726
Dysthymia	77.8	68.7	0.88	0.615
Bipolar disorder	79.5	77.7	0.98	0.877
GAD	68.4	62.1	0.91	0.68
OCD	82.9	84.1	1.01	0.877
Alcohol abuse	96.9	80.5	0.83	0.007
Alcohol dependence	93.2	97.1	1.04	0.469
Any mental disorder	82.1	78.4	0.95	0.385

*Any mental disorder* has at least one of the mental illnesses assessed by the composite international diagnostic interview in the study, *GAD* generalized anxiety disorder, *MDD* major depressive disorder, *OCD* obsessive compulsive disorder

*To compare the prevalence and treatment gap between two surveys, data from SMHS-2016 were reanalysed using similar sampling weight as the SMHS-2010 study; Statistical significance was evaluated at the *p* value < 0.05

^#^Chi-Square test

92 respondents with 12-month diagnosis of any mental disorder reported that at some point during the past 12 months, they had felt the need to see a professional for their problems with nerves, emotions, mental health or their use of alcohol or drugs. Of these 92 respondents, 50 reported thinking that they might need professional health for at least a period of 4 weeks. The reasons for not seeking treatment are shown in Table 5. The reasons most endorsed by the participants for not seeking help despite their feeling that they needed for help were ‘concerns regarding cost of treatment’ followed ‘the problem went away by itself and they did not really need help’.

**Table 5:** Reasons for not seeking help among respondents with mental illnesses in SMHS 2016

Reasons for not seeking help^a^	*N*	Weighted (%)
My health insurance would not cover this type of treatment	13	18.8
The problem went away by itself, and I did not really need help	26	70.3
I thought the problem would get better by itself	14	47.7
I was concerned about how much money it would cost	16	74.6
I was unsure about where to go or who to see	14	64.5
I did not think treatment would work	5	15.1
I was concerned about what others might think if they found out I was in treatment	8	43.1
I thought it would take too much time or be inconvenient	7	22.4
I wanted to handle the problem on my own	13	64.9
I could not get an appointment	1	3.2
I was scared about being put into a hospital against my will	7	43.8
I was not satisfied with available services	4	15.3
I received treatment before and it did not work	5	18.3
The problem did not bother me very much	3	8.1
I had problems with things like transportation, childcare, or scheduling that would have made it hard to get to treatment	7	20.9

^a^Participants were allowed to endorse more than one reason

## Discussion

The prevalence of overall 12-month treatment gap was 78.6%. While this is a significant treatment gap, it is not very different from those reported elsewhere. Data from the World Mental Health Japan (second) survey estimated a treatment gap of 78.1%, reporting that among the respondents with any 12-month mental disorder, only 21.9% had sought treatment [28]. Similarly, the Bern Epidemiological At-Risk (BEAR) study found that among participants reporting any current axis-I disorder, 48.7% reported having sought help for a mental problem at any time; 35.1% reported having sought help in the past and 13.6% were currently help-seeking [20].

The most frequently endorsed barriers to help-seeking were concerns regarding the cost of treatment, and not needing help as the problem went away by itself. Interestingly, about 40% of people did not seek help as they were worried about what others may think if they found out they were in treatment. Our findings are similar to that reported by Mojtabai [29] who examined barriers to help-seeking among participants with major depressive disorder in the National Survey on Drug Use and Health, USA (NSDUH 2005 and 2006). Cost of treatment and the worry that others may find out about their condition were significant concerns among those not seeking treatment. The cost of mental health treatment varies across sectors in Singapore. Restructured hospitals that provide most of the specialist care do provide subsidies to those who cannot afford treatment [30]. However, it is possible that people are not aware of how much it would cost as length of treatment, types of services needed and the need for specialist care (versus primary health care) are difficult to determine for the person who needs treatment. Until recently, there was no insurance coverage for mental illness in Singapore which may have further heightened the cost concerns of those with mental illness [31]. Reducing the cost barriers, both by increasing awareness of the types of treatments offered and their costs, right siting of care through community mental health services and improving the quality of mental health treatments in primary care settings remain important challenges for mental healthcare delivery. Two other frequently endorsed reasons were attitudinal barriers, i.e., that the problem got better by itself and that they could handle it on their own. These findings are similar to that of Sareen et al. [32] who examined perceived barriers to service utilization across the USA, Canada and Netherlands. Remission of mental disorders does occur without treatment and the desire to solve the problem on their own may reflect mild or transient distress; however, untreated illness can lead to persistence of illness, increasing severity and poorer outcomes over time. Such statements may, thus, be indicative of both a lack of mental health literacy and self-reliance which is a commonly endorsed reason among young adults for not seeking help [33]. This coupled with the acknowledgement that 64.5% did not know where to seek help suggests the need for promoting mental health literacy, especially the recognition of signs and symptoms of mental illness and places where they could seek help. Addressing these needs while respecting self-reliance may be possible by creating awareness of evidence-based self-help strategies including sources of e-mental health [34] given that Singapore is a digitally advanced country.

Treatment gap varied across disorders; alcohol dependence had the highest treatment gap (97%), while the lowest treatment gap was observed among those with GAD (62.1%). Similar differences have been reported in other studies [19, 20]. Several factors could have contributed to the high gap observed amongst those with alcohol dependence. A previous nationwide study in Singapore reported that alcohol use disorder was the most stigmatized condition with people endorsing the perception that those with alcohol dependence are more harmful/dangerous and expressing their desire for social distancing from such a person [10]. The stigma against the condition coupled with the perception that it is a social problem and unawareness of the effectiveness of medical treatment is likely to have contributed to the treatment gap. Edlund et al. [35] suggested that people’s perception of need for treatment is a significant determinant of the large treatment gap in alcohol use disorder. While failure to perceive need was found to be the major reason for individuals with alcohol use disorders not receiving treatment, the majority among those with perceived need received treatment across two national surveys examining alcohol and substance use in the United States of America. OCD had the second highest treatment gap in the current study and associated with the high prevalence makes it a significant public health concern. A vignette-based study of mental health literacy found that recognition of OCD in Singapore was much lower as compared to MDD (28.7% versus 55.2%) and about 15% of respondents felt that the person described in the vignette did not have a problem [8]. This may have led to lack of treatment seeking in this group. However, an alternative explanation for the large treatment gap could be that CIDI 3.0 is picking up false-positive cases of OCD and true treatment gap may, thus, be lower. However, as we have reported in a previous article, the high 12-month prevalence of OCD seems consequent to the high persistence of the disorder which in fact may be as a result of the failure to seek treatment [23].

The determinants of treatment gap are unique to our study and are reflective of Asian culture and values. One interesting finding is that despite cost being cited as a barrier, we found that those with lower education (primary and below) and those who were unemployed were more likely to seek treatment. In Singapore, meritocracy is highly regarded, and this is reflected in its merit-based public education and competitive scholarship system. Those with higher education would want to conform to societal expectations, i.e., that they should be resilient to stressors and seen to be high functioning, and thus may prefer not to seek treatment—a reflection of ‘label avoidance’ as mental disorders are widely perceived as a weakness and not illness in the society [10, 36]. Those with higher education may also be fearful that knowledge of their mental disorder in the educational or work setting may affect their future prospects. Interestingly, the Japanese world mental health survey also found that those who were unemployed were more likely to receive treatment. This could be due to two reasons—either those with a more severe disorder and impairment of functioning were unemployed, and thus were more willing to seek treatment or those who were employed were concerned about the consequences of disclosure, and hence did not seek treatment. The Tripartite Guidelines on Fair Employment Practices in Singapore [37] emphasizes the importance of recruiting and selecting employees on the basis of merit, regardless of their background; however, employers do ask potential employees to declare pre-existing medical conditions which includes psychiatric conditions. Anecdotally, mentally ill patients often express their unwillingness to do so as they fear this may adversely affect their employability. While there is a paucity of studies on employer attitudes in Singapore, a study of 550 employers in the UK by the Shaw Trust found that 56% of employers were reluctant to employ someone with a mental health condition due to fear of them being stigmatized by their co-workers and half of them viewed employing individuals with mental health conditions as a ‘significant risk’ to their business [38]. Similarly, people with a mental disorder are reluctant to reveal it to their employers fearing damage to their career, and losing workplace friends [39]. These factors may lead to avoidance of treatment as they may fear inadvertent disclosure at the workplace.

Surprisingly, those with a comorbid chronic physical condition were less likely to have sought treatment. Clinicians treating the physical health condition may miss or underestimate the extent of the psychiatric condition. The demands of managing the chronic physical condition may lead to omission of symptoms of mental disorders (both by provider and patient) during the clinic visits. Clinicians may also attribute nonspecific symptoms, such as fatigue or poor concentration to the physical illness. Even when they recognize the symptoms, they may assume that the distress experienced by the person may be treated if the physical condition is well managed [40, 41]. On the other hand, patients may be struggling to manage the chronic physical condition which may be time and resource intensive, thus leading to neglect or disregard of symptoms of mental disorder.

While the treatment gap of all disorders except OCD and alcohol dependence had decreased from that reported in the earlier 2010 survey [22], the only significant difference was observed among those with alcohol abuse. It must be acknowledged that the demonstration of the effectiveness of interventions implemented at the population level takes time and the decreasing trends are promising. The early years of the master plan were focused on the development of service models and establishment of processes prototyping, while large-scale expansion of the initiatives only started in 2015. On the other hand, it is also possible that the community mental health master plan, while increasing access to community mental health services did not adequately target deep-rooted concerns like stigma and lack of mental health literacy may need to be addressed before one can see a paradigm shift in the attitudes towards help-seeking among those with mental disorders. To address the treatment gap among the employed, it would also be necessary to advocate and work with employers to change hiring practices and put in place a mental health support framework in the workplace for employees. Schools and institutions of higher learning should be cognizant of the importance of the need for mental health education and awareness as most mental disorders have an early age of onset with the majority emerging during adolescence and early adulthood [42–44]. Help-seeking, however, remains low in this population leading to treatment delay and adverse outcomes [45]. Educational institutions, thus, need to include mental health education as an integral part of their curriculum and ensure the implementation of other measures like peer-support initiatives, and online counseling and therapy to enable easy access for students to treatment in a safe and confidential environment [46, 47].

Some limitations of the current study need to be acknowledged. The cross-sectional design of our study is a limitation which is common among many epidemiological studies; this prevented us from examining causal associations. The study did not include a clinical validation of the CIDI 3.0 diagnosis. It is difficult to eliminate recall bias and reporting errors from cross-sectional and self-reported data. While we focused on only past 12-month treatment gap, there could still be discrepancies. Ethical concerns and personal data protection laws do not allow linkage to hospital administrative data and hence we are unable to determine if there was any under or over reporting of the treatment gap. The survey did not include those institutionalized throughout the survey period. Though excluded people comprised a small proportion of the population, their exclusion could have some effect on the reported treatment gap. While the survey had a reasonably good response rate (69.5%), it is possible that those refusing to participate in the study had a higher prevalence of mental disorders or had pre-existing mental disorders that were not treated. Lastly, the survey did not include information on pharmacotherapy and treatment duration; thus, we could not assess treatment adequacy in the population.

The strengths of the study include the large sample size, ensuring consistency of methodology across both surveys and conduction of interviews in multiple local languages to ensure inclusivity.

In conclusion, our study found a significant treatment gap among people with mood, anxiety and alcohol use disorder in Singapore which is consistent with previous research. The extent of treatment gap has decreased since the last study, but it was not significant except in the case of alcohol abuse, suggesting the early effects of the community mental health master plan in improving the treatment gap. The study identified some correlates of treatment gap which are unique to the Asian context. Our findings suggest that a number of barriers, namely, individual (financial, attitudinal, lack of awareness) or cultural (stigma), may contribute to the treatment gap. These barriers need to be addressed by different, culturally appropriate health policies and interventions including promotion of school based and workplace mental health initiatives and implementing anti-stigma campaigns. These interventions must be made available in all local languages, incorporate locally relevant concepts and content, and provide referrals to appropriate mental health services as well as to e-health sources that are adapted for use in Singapore.

## Electronic supplementary material

Below is the link to the electronic supplementary material.

Supplementary material 1 (RTF 95 kb)
