# Supplementary material for: Minding the treatment gap: results of the Singapore Mental Health Study
Source: Soc Psychiatry Psychiatr Epidemiol. 2019 Jul 17;55(11):1415–24. doi: 10.1007/s00127-019-01748-0 (PMC7578124; doi:10.1007/s00127-019-01748-0)
Supplement: Supplementary file 1 — Supplementary material 1 (RTF 95 kb) [file 127_2019_1748_MOESM1_ESM.rtf]

Supplementary Table 1: Association of Sociodemographic Variables with Treatment Gap*
	Treatment gap		
	       No	      Yes		
	n	%	n	%	P value#	
Education						
Post-secondary	29	15.7	132	84.3	0.0867	
Primary and below	15	39.6	26	60.4		
Secondary	31	21.2	170	78.8		
Employment						
Employed	51	18.1	238	81.9	0.0359	
Economically inactive	9	21.2	60	78.8		
Unemployed	15	43.1	30	56.9		
Severity 						
Mild	29	16.5	202	83.5	0.0155	
Moderate/Severe	41	32.9	96	67.1		
12-month CIDI						
MDD 	17	26.6	72	73.3	0.0076	
Dysthymia	2	38.6	3	61.3		
Bipolar	8	22.3	30	77.7		
GAD	12	48.1	17	51.8		
OCD	12	11.7	112	88.3		
AUD	1	0.4	35	99.6		
Comorbidity	23	26.9	59	73.1	0.0076	
*Only significant associations are shown
# Chi-Square analyses
